# Genetically encoded calcium indicators for fluorescence imaging in the moss *Physcomitrella*: GCaMP3 provides a bright new look

**DOI:** 10.1111/pbi.12769

**Published:** 2017-07-20

**Authors:** Thomas J. Kleist, Heather N. Cartwright, Adele M. Perera, Michael L. Christianson, Peggy G. Lemaux, Sheng Luan

**Affiliations:** ^1^ Department of Plant Biology Carnegie Institution for Science Stanford CA USA; ^2^ Department of Plant and Microbial Biology University of California Berkeley Berkeley CA USA; ^3^ Department of Environmental Science Policy & Management University of California Berkeley Berkeley CA USA

**Keywords:** Calcium (Ca^2+^), biosensor, *Physcomitrella*, GCaMP, Yellow CaMeleon (YC)

Dear Editor,

The moss *Physcomitrella patens* is emerging as a model organism due to the availability of established protocols for precise genome engineering by homologous recombination and a high‐quality genome sequence (Rensing *et al*., 2008). *Physcomitrella* is an attractive system for cell biology and live cell imaging and has been used to investigate fundamental cellular processes such as tip growth, vesicle trafficking and cytoskeletal dynamics (e.g., Furt *et al*., 2012). In each of these processes, calcium (Ca^2+^) acts as an important signal to relay developmental and environmental cues. Classical techniques have been used to monitor Ca^2+^ dynamics in this early land plant model. Using the luminescent Ca^2+^ reporter aequorin, osmotic shock or mechanical perturbations were shown to elicit transient Ca^2+^ signals in *Physcomitrella* (Haley *et al*., 1995; Russell *et al*., 1996), but this approach provided limited spatial information about these responses. Alternatively, *Physcomitrella* cells have been biolistically loaded with synthetic fluorescent Ca^2+^ indicator dyes to visualize saline‐elicited Ca^2+^ signals (Qudeimat *et al*., 2008); however, this approach damages the cells used for Ca^2+^ imaging and is poorly suited for long‐timescale experiments because of dye sequestration and dilution. To extend the Ca^2+^‐imaging toolkit for *Physcomitrella*, we engineered lines transformed with genetically encoded calcium indicators (GECIs) and assessed their performance using conditions known to trigger transient Ca^2+^ changes.

Two GECIs, Yellow Cameleon 3.6 (YC3.6) (Nagai *et al*., 2004) and GCaMP3 (Tian *et al*., 2009), were selected for *in vivo* evaluation in *Physcomitrella*. At their core, each sensor contains an engineered Ca^2+^‐binding calmodulin (CaM) variant and CaM‐binding peptide fused to one or two fluorophores that optically report Ca^2+^‐dependent conformational changes. The YC3.6 sensor contains a cyan fluorophore (enhanced cyan fluorescent protein, eCFP) and a yellow fluorophore (cpVENUS), which can accept energy from eCFP through Förster Energy Resonance Transfer (FRET); FRET magnitude is positively correlated with Ca^2+^ concentration. The more recently developed GCaMP3 sensor contains a single circularly permuted green fluorescent protein (cpGFP); and fluorescence intensity (FI), which is positively correlated with Ca^2+^ concentration, is directly used as the signal readout. FRET‐based GECIs such as YC3.6 have been widely used in higher plant systems, however, GCaMP3 and related GECIs reportedly offer superior performance *in vitro* and in animal systems (reviewed by Rodriguez *et al*., 2017). In *Arabidopsis thaliana*, R‐GECO1, which was engineered from GCaMP3 by replacing cpGFP with the red fluorophore cpmApple (Zhao *et al*., 2011), has been reported to offer greater response magnitude compared to YC3.6 (Keinath *et al*., 2015). To our knowledge, there are no publications so far that report similar comparisons using GCaMP3 or related green GECIs in any plant or that describe use of fluorescent GECIs in a bryophyte.

To engineer *Physcomitrella* lines expressing fluorescent GECIs, protonemal cells were cultured on cellophane‐overlaid agar growth media and bombarded with DNA‐coated gold particles. Expression of YC3.6 or GCaMP3 was driven by a *Zea mays Ubiquitin1* promoter with its 5′ leader intron. Because GCaMP3 is an intensiometric sensor, we co‐transformed GCaMP3 lines with *Porites porites* red fluorescent protein (RFP) driven by a *Panicum virgatum Ubiquitin1* promoter to serve as an internal reference and control. Fluorescent signal could be detected 48 h after bombardment (Figure S1), and transformed cells were selected by antibiotic resistance. Colonies that grew under the first round of selection were embedded in antibiotic‐supplemented media and maintained under sterile conditions in growth chambers (Appendix S1). This method has allowed us to rapidly generate populations of stably transformed *Physcomitrella* lines that have been maintained over 3 years (to date) by periodic subculture.

Transgenic lines expressing YC3.6 were prepped for Ca^2+^ imaging by growing small colonies on cellophane‐overlaid plates and transferring them to coverslips with media reservoirs (Appendix S1). Spinning disc confocal microscopy was used to collect emissions from the donor fluorophore under direct excitation (DxDm), the acceptor fluorophore under donor excitation (DxAm) and the acceptor fluorophore under direct excitation (AxAm). Salinity stress was applied by stepwise increases in NaCl concentration (Figure 1a, Movies S1). Each addition of NaCl provoked a transient (2–3 min) increase of approximately 3%–7% in DxAm FI across the field of view and a corresponding decrease of similar magnitude in DxDm FI (Figure 1b). At approximately 350 mm NaCl, cells plasmolysed, and FIs decreased by approximately 40% in each channel. The control treatment using media lacking NaCl did not elicit FI changes that could be discriminated from background noise; thus, the YC3.6 sensor is suitable for monitoring salinity‐elicited Ca^2+^ signalling events in *Physcomitrella*. When sensor readout is plotted as the DxAm/DxDm ratio, there is a clear decline in the DxAm/DxDm ratio: the initial resting‐state value of 1.25 gradually falls to 1.10—roughly a 14% decrease—just before the final treatment leading to plasmolysis. We also observed a ~14% decrease in the AxAm channel over the ~37 min experiment; therefore, the decrease in ratio is likely due to acceptor photobleaching. Further experimentation may allow for improved postacquisition data processing; however, we decided to test whether GCaMP3 may offer superior performance without requiring substantial postacquisition analysis.

**Figure 1:**
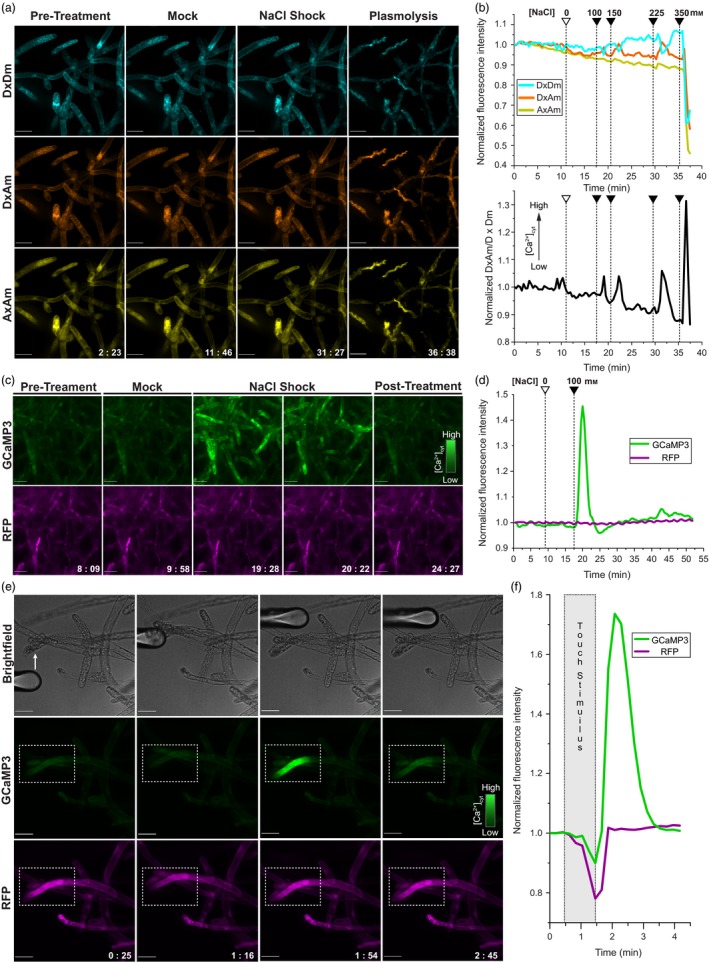
(a) Imaging saline‐elicited Ca^2+^ signals in *Physcomitrella* using YC3.6. Rows show fluorescent signal from the donor fluorophore under donor excitation (DxDm), acceptor fluorophore under donor excitation (DxAm) and acceptor fluorophore under acceptor excitation (AxAm). Columns show images taken pretreatment, following mock control treatment, following NaCl shock (225 mm total) and after plasmolysis at 350 mm NaCl. See Movie S1. Fluorescence images are average z‐stack projections of spinning disc confocal data. Scale bars indicate 50 μm; timestamps are in mm:ss format. (b) Quantitative summary of YC3.6 salinity stress treatments. Mock control or salt shock treatments (empty/filled arrowheads) are indicated alongside total concentration of NaCl in the reservoir. (*top*) Normalized mean FIs for the DxDm, DxAm and AxAm channels across the entire field of view. (*bottom*) Normalized ratio of the same DxAm/DxDm data, which is positively correlated with cytosolic Ca^2+^ concentration ([Ca^2+^]_cyt_). (c) Imaging saline‐elicited Ca^2+^ signals in *Physcomitrella* using GCaMP3. Top row of images shows GCaMP3 signal; bottom row shows RFP signal. Cells are shown pretreatment, during mock control, during NaCl shock and post‐treatment. See Movie S2. (d) Quantitative summary of GCaMP3 saline treatment. Normalized mean FIs for the GCaMP3 (green) and RFP (magenta) channels are shown. The mock control and salt shock treatment (empty/filled wedges) were applied at the indicated points. Total NaCl concentration in the reservoir is labelled. (e) Touch‐elicited Ca^2+^ signals in *Physcomitrella* imaged using GCaMP3. Top row shows midplane brightfield images, followed by GCaMP3 and RFP signals, respectively. Dotted box indicates region of interest (ROI) used for quantitation. See Movie S3. (f) Quantitative summary of touch‐elicited Ca^2+^ signals. Shaded box indicates duration of touch stimulus.

Transgenic lines expressing GCaMP3 and the internal reference RFP were prepped for salt shock experiments using the same procedure as YC3.6 lines. After a mock control, which was performed by addition of media lacking NaCl and did not elicit signal intensity changes, cells were treated with 100 mm NaCl. The treatment elicited a pronounced spike in GCaMP3 FI, whereas RFP signal intensity remained steady throughout the experiment (Figure 1c, Movie S2). At its peak, which occurred approximately 2 min after the initial salt shock, the GCaMP3 FI increased by ~45% across the field of view. Signal returned to baseline intensity within 5 min of treatment without removal or dilution of NaCl (Figure 1d). We did not encounter noticeable photobleaching of GCaMP3 or RFP over the course of the ~50‐min experiment. Our data collectively suggest that GCaMP3 is a simple, reliable GECI to use in *Physcomitrella* that offers advantages over YC3.6 in terms of photostability and response magnitude and requires less postacquisition data processing.

The striking performance of GCaMP3 in *Physcomitrella* protonemal cells during salt shock assays prompted us to monitor mechanically evoked Ca^2+^ signals in this system. Mechanical responses in cells expressing GCaMP3 were assayed by stimulation with a round‐tipped glass microprobe mounted on a motorized micromanipulator. Transient elevations in GCaMP3 FI were elicited by touching cells with the microprobe and were locally confined near the stimulated region (Figure 1e,f; Movie S3). Similar FI increases were not observed in the RFP channel. Mere movement of the probe next to cells, without making contact, did not elicit this response, implying that mechanical stimulation was responsible for observed Ca^2+^ transients. This simple demonstration elegantly reveals subcellular Ca^2+^ dynamics associated with mechanical stimuli and suggests that GCaMP biosensors may be useful tools to investigate plant mechanoreception.

In this study, we engineered transgenic *Physcomitrella* lines expressing fluorescent biosensors YC3.6 or GCaMP3 and validated their utility for Ca^2+^ imaging, while highlighting the advantages of GCaMP3. Combined with currently available molecular genetic tools for *Physcomitrella*, the simple and rapid method for biosensor deployment presented here provides a promising approach for phenotypic interrogation of mutant lines. Going forward, this method can be used to engineer moss lines that express other GECIs, such as the more newly developed GCaMP6 series, or biosensors for other molecules, such as metabolites or hormones.

## Conflict of interest

The authors declare no conflict of interest.

## Supporting information


**Figure S1** Fluorescent signal from *Physcomitrella* cells successfully transformed with RED FLUORESCENT PROTEIN (RFP) using particle bombardment.

**Appendix S1** Materials & Methods.

**Movie S1** Imaging saline‐elicited Ca^2+^ signals in *Physcomitrella* using the FRET‐based sensor YC3.6.

**Movie S2** Imaging saline‐elicited Ca^2+^ signals in *Physcomitrella* using GCaMP3.

**Movie S3** Imaging touch‐elicited Ca^2+^ signals in *Physcomitrella* using GCaMP3.
