# Supplementary material for: Genetically encoded calcium indicators for fluorescence imaging in the moss Physcomitrella: GCaMP3 provides a bright new look
Source: Plant Biotechnol J. 2017 Jul 20;15(10):1235–7. doi: 10.1111/pbi.12769 (PMC5595717; doi:10.1111/pbi.12769)
Supplement: Supplementary file 2 — Appendix S1 Materials & Methods. [file PBI-15-1235-s005.docx]

**Supplemental Information SI1: Materials & Methods**

**Growth media.**

*Physcomitrella* was grown in BCDAT medium, as described previously (e.g., Nishiyama *et al*., 2000), solidified with 0.8% Phytoblend (Caisson Labs). Cellophane disks (AA Packaging LTD) were sterilized by autoclave before application to solidified media. Cultures were maintained as protonema by weekly immersion blending in liquid BCDAT medium using an IKA T-18 ultra-turrax homogenizer equipped with a S 18 N – 19 G dispersing element. Micropore tape (3M Company) was used to seal growth plates to maintain sterility.

**Growth conditions.**

*Physcomitrella* was grown under cool white fluorescent bulbs at approximately 120 microeinsteins with a 16 hour day / 8 hour night cycle. Temperature was maintained between 22 – 24 degrees Celsius.

**Molecular cloning.**

The vector containing YC3.6 cloned under the regulatory control of the *Zea mays UBIQUITIN1* promoter was kindly provided by the lab of Prof. Giles Oldroyd (John Innes Center). The coding sequence of the GCaMP3 calcium indicator was subcloned by Gateway (Thermo Fisher) LR reaction into pANIC5A plant expression vector (Mann et al., 2012) and was driven by the *Zea mays* *UBIQUITIN1* promoter. The pANIC5A vector contains a second FP, *Porites porites* RFP, under the control of the switchgrass (*Panicum virgatum*) *POLYUBIQUITIN1* promoter. Each vector contains the selective marker HYGROMYCIN PHOSPHOTRANSFERASE (HPH) driven by an *Oryza sativa ACTIN1* promoter, including its 5’ leader intron.

**Microscopy.**

Stereofluorescence images showing protonemal cells post-bombardment were acquired using a Zeiss Stereo Lumar (Carl Zeiss) equipped with a QImaing 3 MPix digital color camera using a Texas Red filter set. Images were collected on a Leica DMI6000B equipped with Yokogawa CSU-X1. For YC3.6 experiments, samples were excited with a 442 nm (donor excitation, Dx) or 514 nm laser (acceptor excitation, Ax), and 442/514 dichroic (Semrock) was used. Under donor excitation, donor emissions (DxDm) were collected from 454-496 nm, and acceptor emissions (DxAm) were collected from 533-547 nm. Acceptor emissions were collected in the same range under acceptor excitation (AxAm). For GCaMP3 experiments, samples were excited alternately using 488 nm or 561 nm lasers. A 488/561 dichroic was used. GFP (GCaMP3) signal was collected from 500-550 nm, and RFP signal was detected from 573-736 nm. In each case, a Leica HC PL APO 20x/ 0.7 IMM CORR objective was used with glycerin. Emissions were collected using a Photometrics Evolve 512 EM CCD camera. Images were acquired and analyzed using Slidebook version 6 (Intelligent Imaging Innovations). Images and movies shown are average z-stack projections of confocal data. Samples were mounted on Brain Research Laboratories 48x60 mm cover slips (Cat. #4860-1-1/2) in small reservoirs made using silicone vacuum grease and filled with ½ strength liquid BCDAT media. For mechanostimulation, a Sutter Instruments model P-97 micropipette puller was used to craft a round-tip glass probe that was mounted on a Sutter Instruments motorized 3-axis micromanipulator controlled by a Sutter Instruments MP-285 unit. For sodium chloride (NaCl) stress treatments, a droplet of ½ strength BCDAT with or without 2 M NaCl was added to the reservoir, and the total final concentration is listed. Each experiment was repeated at least 6 times with consistent results.

**SI1 References**

Mann, D.G., LaFayette, P.R., Abercrombie, L.L., King, Z.R., Mazarei, M., Halter, M.C., Poovaiah, C.R., Baxter, H., Shen, H., Dixon, R.A., and Parrott, W.A**.** (2012). Gateway‐compatible vectors for high‐throughput gene functional analysis in switchgrass (*Panicum virgatum* L.) and other monocot species. *Plant Biotechnology Journal*, **10**, 226-236.

Nishiyama, T., Hiwatashi, Y., Sakakibara, K., Kato, M. and Hasebe, M. (2000). Tagged mutagenesis and gene-trap in the moss, *Physcomitrella patens* by shuttle mutagenesis. *DNA Research*, **7**, 9-17.

**Step-by-step biolistic transformation protocol for *Physcomitrella*.**

1. Prepare sterile cellophane-overlaid BCDAT media plate and apply freshly blended protonemal culture; dry onto cellophane in sterile flow hood.

*Note*: Only a thin layer of protonema is needed for efficient transformation;

selection can be hampered by excess cell density.

1. Place cultures in growth chamber in the light, wait 2-3 days for protonema to recover from blending.
2. Prepare and sterilize Bio-Rad PDS-1000/He bombardment chamber and components.
3. Vortex 1 micron DNAdel gold particles (Seashell Technologies) at maximum velocity for > 5 minutes.
4. Add manufacturer-supplied binding buffer to gold particles at 2:3 volume ratio of buffer to particles; vortex 30 seconds.
5. Add transforming DNA (pre-adjusted to 1,100 ng/μL) at 1:6 volume ratio of DNA to particles; vortex 30 seconds.
6. Add an equal volume of manufacturer-supplied precipitation buffer (equal to total volume of particles + buffer + DNA) dropwise to solution containing particles while vortexing.

*Note*: Keep tube upright while vortexing to avoid losing particles or solution.

1. Close tube and vortex vigorously; allow to sit upright for 3 minutes at room temperature.
2. Centrifuge at 10,000 x g for 10 seconds.
3. Remove supernatant, wash particles by addition of 500 μL ice-cold 100% ethanol; vortex > 3 minutes.
4. Centrifuge at 10,000 x g for 10 seconds.
5. Resuspend particles in ice-cold 100% ethanol, using 2x the original volume of gold; vortex > 3 minutes.
6. Apply 10 μL of particle suspension to each macrocarrier disk (pre-loaded into macrocarrier holding rings).
7. Load sterilized Bio-Rad 1,100 PSI rupture disk into bombardment chamber.
8. Load macrocarrier holding shelf at the top position in the bombardment chamber (disk-macrocarrier gap distance = 6.4 mm; macrocarrier travel distance = 8 mm).
9. Load plates containing protonema at the second lowest position in the bombardment chamber (target distance = 9 cm).
10. Bombard each plate once. Bring vacuum to a pressure of ~640 mmHg before shot.
11. Allow protonema to recover from bombardment for 1-2 days in the light.

*Note*: Fluorescent marker signal can be checked to validate transformation.

1. Move cellophane containing protonema onto selective media.

*Note*: 50 μg / mL Hygromycin B (Thermo Fisher, Cat. 10687010) can be used effectively for the Pro_OsAct1_::HPH marker.

1. After 2 weeks on selective media, pick regenerating colonies and transfer to fresh selective media.
