# Supplementary material for: Genetically encoded calcium indicators for fluorescence imaging in the moss Physcomitrella: GCaMP3 provides a bright new look
Source: Plant Biotechnol J. 2017 Jul 20;15(10):1235–7. doi: 10.1111/pbi.12769 (PMC5595717; doi:10.1111/pbi.12769)
Supplement: Supplementary file 6 — Legends [file PBI-15-1235-s006.docx]

**Supplemental Figure Legend SF1: Fluorescent signal from *Physcomitrella* cells successfully transformed with RED FLUORESCENT PROTEIN (RFP) using particle bombardment.**

Protonemal cells 48 hours post-bombardment. Brightfield (BF) image is shown on the left, and red fluorescent protein (RFP) signal is shown on the right. Red-orange foci are indicative of successfully transformed cells.

**Supplemental Movie Legend SM1: Imaging saline-elicited Ca^2+^ signals in *Physcomitrella* using the FRET-based sensor YC3.6.**

Moss protonemal cells expressing the ratiometric Ca^2+^ indicator YC3.6 were monitored during application of a mock or saline stress treatments. Treatments are indicated by text titles, and time stamp is in mm:ss format. Each movie is a z-stack projection of spinning disk confocal data. Channel labels are described in legend for Figure 1A-B.

**Supplemental Movie Legend SM2: Imaging saline-elicited Ca^2+^ signals in *Physcomitrella* using GCaMP3.**

Moss protonemal cells expressing the Ca^2+^ indicator GCaMP3 (green) and an internal visual reference, RFP (magenta), were monitored during application of a mock or saline stress treatment at indicated marks. Each movie is a z-stack projection of spinning disk confocal data. Movie is an average z-stack projection of spinning disk confocal data. Timestamp is shown in mm:ss format. Channel labels are described in legend for Figure 1C-D.

**Supplemental Movie Legend SM3: Imaging touch-elicited Ca^2+^ signals in *Physcomitrella* using GCaMP3.**

Moss protonemal cells expressing the Ca^2+^ indicator GCaMP3 (green) and an internal visual reference, RFP (magenta), were mechanically stimulated with a glass microprobe driven by a motorized micromanipulator. The average z-stack projection of each channel is shown except for the brightfield channel, which was acquired at midplane only. Timestamp is shown in mm:ss format. brightfield illumination). Channel labels are described in legend for Figure 1E-F.
